# Patient–Caregiver Treatment Preference Discordance and Its Association With Caregiving Burden and Esteem

**DOI:** 10.1093/geroni/igab020

**Published:** 2021-06-14

**Authors:** Semra Ozdemir, Sean Ng, Chetna Malhotra, Irene Teo, Eric A Finkelstein, COMPASS Study Group, Ratna Singh, Rebecca A Dent, Wee Lee Yeo, Yin Bun Cheung, Rahul Malhotra, Ravindran Kanesvaran, Alethea Chung Pheng Yee, Noreen Chan, Huei Yaw Wu, Soh Mun Chin, Hum Yin Mei Allyn, Grace Meijuan Yang, Patricia Soek Hui Neo, Nivedita V Nadkarni, Richard Harding

**Affiliations:** 1 Lien Centre for Palliative Care, Duke–NUS Medical School, Singapore; 2 Signature Programme in Health Services and System Research, Duke–NUS Medical School, Singapore; 3 National Cancer Centre Singapore, Singapore

**Keywords:** Caregiver stress, Death and dying, End-of-life care, Palliative care, Quality of care

## Abstract

**Background and Objectives:**

Many patient–caregiver dyads report conflicting treatment decisions regarding preferences for life extension treatments and symptom management. It is possible that this discordance will lead to negative psychological outcomes including lowered caregiving esteem and increased caregiver burden. However, the relationships between treatment discordance among dyads and caregiver psychological outcomes are not well studied among advanced cancer patients—a gap this study aims to fill.

**Research Design and Methods:**

Outcome variables included caregiver burden and caregiving esteem, measured via a modified 4-domain Caregiver Reaction Assessment Scale. The main independent variable was patient–caregiver treatment preference discordance, examined using questions adapted from an existing protocol. Analyses were conducted using multivariable regressions.

**Results:**

A convenience sample of 285 patient–caregiver dyads were recruited from outpatient clinics at 2 tertiary hospitals in Singapore. The majority (60%) of patient–caregiver dyads reported discordant treatment preferences. Discordance in this study arose because caregivers wanted a balance between life extension and symptom management while patients preferred life-extending treatment. In multivariable analyses, discordance predicted caregiver burden arising from impact on caregiver schedule and health (β = 0.16, *p* = .07) and lack of family support (β = 0.13, *p* = .04).

**Discussion and Implications:**

Theoretically, this study provided a more nuanced understanding of how dyad discordance may worsen the burdens felt by caregivers, and which aspects of their lives (i.e., burden due to impact of caregiver schedule and health and lack of family support) are most affected. Our findings can aid in establishing therapeutic interventions targeted toward improving communication skills and encouraging end-of-life discussions among patients, caregivers, and their health care providers. The importance of establishing and improving therapy programs specifically targeted toward caregivers was also underlined.

**Translational Significance:** Discordance among patient–caregiver dyads in treatment decisions at the end of life may increase caregiver burden. Caregivers who had discordant treatment preferences with their patients reported higher perceived caregiver burden arising from the impact on caregiver schedule and health and lack of family support. Therapeutic interventions targeted to improving communication skills and encouraging end-of-life discussions may reduce discordance and in this way promote caregiver well-being.

## Background and Objectives

Patients with cancer suffer from severe health consequences including acute pain, constant fatigue, and extreme weight loss (Chwistek, 2017; Cooper et al., 2015; Kumar, 2011; Theobald, 2004). For those whose illness progresses to advanced, and often terminal stages, difficult medical decisions must be made. For many, this means the unpleasant choice between balancing treatments that would extend their lives against those of symptom management (Kaur & Mohanti, 2011). Understandably, a significant proportion of cancer patients prefer to not make these decisions alone, with many involving their family caregivers or even deferring decisions to them completely (Pardon et al., 2010; Sekimoto et al., 2004; Shin et al., 2013).

Caregiver involvement in decision making can come with numerous benefits (Shay & Lafata, 2015). However, problems occur when patient–caregiver dyads report conflicting treatment preferences. The issue appears to be pervasive, with estimates indicating that a quarter to half of advanced cancer patients perceived their caregivers’ preferences for end-of-life treatment to be different from their own (George et al., 2019; Phipps et al., 2003). Patients often did not wish to be financial, social, or emotional burdens to their families and were less likely to want aggressive and costly treatments that extended life (i.e., do-not-resuscitate orders, tube feeding; Ozdemir et al., 2019; Tang et al., 2005; Tsai et al., 2015). Contrarily, caregivers were more likely than patients to have preferences toward aggressive life-extending treatments (Ozdemir et al., 2019; Tsai et al., 2015).

It is possible that the discordance regarding end-of-life treatment preferences can overwhelm caregivers and lead to many negative psychological outcomes including lowered caregiving esteem (i.e., the self-esteem that is associated with caregiving). It may also be associated with an increase in caregiver burden—a multidimensional construct capturing the perceived physical, emotional, and financial stresses resulting from caregiving (Bastawrous, 2013). To the best of our knowledge, however, these associations have not been well studied. The only evidence is shown by Tsai et al. (2015) indicating that levels of perceived caregiver burden were positively associated with disagreements regarding treatment preferences between patients with dementia and their caregivers, particularly for end-of-life preferences. However, the focus of the researchers was on dementia—a disease that differs significantly from cancer in terms of illness trajectories, caregiving responsibilities, and treatment options (Birner et al., 2016; Duong et al., 2017; Scheel & Holtedahl, 2015; Scott & Barrett, 2007). It remains unclear whether results can be generalized to cancer. Tsai et al. also examined caregiver burden as a construct with a single domain. However, as caregiver burden affects many facets of the caregivers’ lives including their social, emotional, and financial health, it is important that research examines how discordance in treatment preferences may be associated with different domains of caregiver burden. This is in line with the theoretical foundations of the stress process model, which highlights the multidimensional nature of the factors predicting burden. These can include caregiving-related factors, dyad relationships, and the sociodemographic factors of caregivers and those under their care (Conde-Sala et al., 2010; Kim et al., 2012). Evidence on the associations between dyad discordance and caregiving esteem is also limited, an important omission given the inverse associations between caregiving esteem and caregiver distress (Kim et al., 2007).

Based on the literature review, this study aimed to examine the associations between patient–caregiver discordance regarding end-of-life treatment preferences and various dimensions of perceived caregiver burden (i.e., schedule and health, finances, family support) and caregiving esteem. We hypothesized that discordance in treatment preferences would be associated with higher levels of perceived caregiver burden and lower levels of caregiver esteem. The findings from this study will aid in a better understanding of how the psychological health of caregivers may be affected by treatment discordance. By underlining the specific caregiver burden dimensions that are most affected, our findings are also expected to aid in the development of interventions targeted toward improving the psychological health of family caregivers and those under their care.

## Research Design and Methods

### Participants and Setting

This study employed survey data taken from the Cost of Medical Care of Patients with Advanced Serious Illness in Singapore (COMPASS) study—a prospective cohort study examining health care-related variables among Singaporean advanced cancer patients and their family caregivers (Teo et al., 2018). Participants were recruited from outpatient clinics at medical oncology departments of the National Cancer Center and the National University Hospital. For this study, only the baseline data were used.

The inclusion criteria for the recruited patients included being a citizen or permanent resident of Singapore, being at least 21 years old, and having a diagnosis of stage IV solid cancer. To ensure that patients have adequate functional status for participation, only those with an Eastern Cooperative Oncology Group performance status of 2 or less were included. For patients with breast or prostate cancer, an additional criterion of metastasis to an organ site was included. Patients needed to cognitively be able to consent and self-report (determined through medical records or Abbreviated Mental Test for participants aged 60 and older). Caregiver participants were required to be a primary informal caregiver (i.e., those who care for the patient without payment) of the patient defined as one of the main persons who (a) provided care to the patient (i.e., accompanying the patient for doctor’s visits, helping with day-to-day activities), (b) ensured provision of care, and (c) was involved in making treatment decisions on behalf of the patients (Teo et al., 2018).

A research coordinator screened patients from medical records for the inclusion criteria. Individuals who met the inclusion criteria were approached by trained research coordinators. If they agreed to participate, their respective caregivers were approached for recruitment. All participants provided written informed consent. Patients completed surveys that were interviewer-administered while caregivers were given the option of completing theirs on their own. Data from all patient–caregiver dyads were captured electronically using an online survey platform. Ethics approval was obtained from the SingHealth Centralised Institutional Review Board (2015/2781). More information about the study can be found in the COMPASS protocol (Teo et al., 2018).

### Survey Development and Outcomes

Consent forms, surveys, and screeners for all patient–caregiver dyads were administered in their preferred language (English, Mandarin, or Malay). The survey instruments were translated with the aid of a professional translation service with the exception of the scales that already had Mandarin and Malay versions. To ensure the readability of the translated questionnaires, cognitive interviews were conducted with 10 participants in each language (Teo et al., 2018).

#### Patient–caregiver treatment preference discordance

Treatment preferences for the patient–caregiver dyads were assessed through a question adapted from the Cancer Care Outcomes Research and Surveillance Consortium (Malin et al., 2006). Specifically, patients responded to the question, “If you had to make a choice now, would you prefer treatment that extends life as much as possible, or would you want treatment that gives you minimal pain and discomfort?” Similarly, their caregivers answered the question: “If you had to recommend a treatment to (Patient) now, would you recommend a treatment that extends life as much as possible, or would you recommend a treatment that focusses on relieving pain and discomfort as much as possible?” Responses for both questions were measured on a 9-point Likert scale. Patient–caregiver dyads who responded 6–9 on the Likert scale were coded as “1”—preferring minimum life-extending treatment and minimal pain and discomfort (i.e., focus of care is symptom management). Those who chose 5 (the midpoint in the scale) were coded as “2”—having a moderate stance toward both life-extending treatment and symptom management, while individuals who selected options 1–4 were coded as “3”—preferring maximum life-extending treatment, severe pain, and discomfort (i.e., focus of care is life-extending treatment). When treatment preferences on the 1–3 scale differed between patient–caregiver dyads, they were defined as being discordant.

#### Perceived caregiver burden and caregiving esteem

Perceived caregiving burden and caregiving esteem were assessed with the modified Caregiver Reaction Assessment Scale (CRAS)—a reliable measure (Cronbach’s alpha = 0.66–0.82) for examining perceived caregiver burden and caregiving esteem validated for use in a Singaporean population (Malhotra et al., 2012). The CRAS is a four-domain (schedule and health, finances, family support, and caregiving esteem), self-report measure of 21 items measured on a 5-point Likert scale where 1 = “strongly disagree,” 2 = “disagree,” 3 = “neither agree nor disagree,” 4 = “agree,” and 5 = “strongly agree.” Each subscale was scored using the unweighted mean score, which ranged from 1 to 5 (Malhotra et al., 2012). Because the purpose of the CRAS was to examine different dimensions of the impact of caregiving on caregivers’ lives, no overall score was used. A higher score on the caregiving esteem subscale indicated a greater positive caregiving effect, while higher scores on the other subscales (impact on schedule and health, finances, and family support) were indicative of a greater negative caregiving effect (Malhotra et al., 2012).

#### Patient characteristics

In relation to patient characteristics, patient (a) age, (b) gender, (c) education, (d) ethnicity, (e) marital status, (f) availability of private insurance, and (g) level of symptom burden were assessed. To examine the availability of private insurance, patients answered “yes,” “no,” or “don’t know” to the questions “are you covered by an Integrated Shield Plan (i.e., a Singaporean health insurance plan provided by private insurance companies)?” and “do you have private health insurance that helps cover the costs of your medical care?” Patients who answered “yes” to either question were considered to be covered by private insurance. “No” and “don’t know” responses were merged under “no.” Symptom burden was assessed through the summing of patient responses (0 “not at all,” 1 “a little bit,” 2 “somewhat,” 3 “quite a bit,” 4 “very much”) to the presence of nine symptoms over the past 7 days (pain, shortness of breath, constipation, loss of weight, vomiting, swelling in parts of the body, dry mouth and throat, lack of energy, and nausea). The list of symptoms was taken from the Functional Assessment of Chronic Illness Therapy—Palliative Care scale (Lyons et al., 2009). Higher scores were indicative of increased symptom burden.

To examine if patients communicated their treatment preferences with their caregivers, they were asked whether they have discussed their treatment and care preferences with their family member(s). Patients were also asked whether they reported Advance Care Plans/Advance Medical Directives.

#### Caregiver characteristics

We measured caregiver (a) age, (b) gender, (c) education, (d) ethnicity, (e) marital status, (f) working status, (g) self-reported financial status, (h) if they had to provide caregiving to others, (i) number of caregiving hours per week for the patient, (j) relationship to the patient (e.g., wife/husband, son/daughter), and (k) how well they get along with the patient.

To examine if caregivers provided caregiving to anyone other than the patient, we asked the question “do you provide unpaid care for other people in the family?” If a specific number of caregiving hours was provided, this was taken. However, if a range of time was given, the midpoint of the range was taken. To examine how well caregivers got along with the patients, we asked the question “generally, how well do you and (patient) get along together?”

### Statistical Analyses

We first presented either the means (and standard deviations) or frequencies of patient and caregiver characteristics, treatment preferences, and the four CRAS subscales. Chi-square tests were used to investigate if dyad concordance/discordance was associated with dyad communication and advance care planning.

To examine the associations between patient–caregiver dyad treatment preferences and perceived caregiver burden, we first conducted a *t*-test for equality of mean CRAS scores between discordant and concordant dyads. We then ran separate multivariable linear regression models estimated via Ordinary Least Squares where the dependent variables were the scores on each of the four domains of the CRAS. The main independent variable of interest was discordance, where “1” demonstrated the presence of discordance and “0” was indicative of no discordance. These analyses were controlled for patient and caregiver characteristics that past literature associated with caregiver burden. Specifically, for patient characteristics we controlled for age (Lee et al., 2019), symptom burden (Dyck et al., 1999), and availability of private insurance (Hu et al., 2018; Yes = 1, No = 0). Regarding caregiver characteristics, we controlled for age (Brazil et al., 2003), gender (Hsiao, 2010; Male = 1, Female = 0), current marital status (Park et al., 2012; Married = 1, Not Married = 0), working status (Bekdemir & Ilhan, 2019; Working Full Time or Part Time = 1, Not Working = 0), self-reported financial status (Bradley et al., 2009; Adequate and Above = 1, Below Adequate = 0), relationship to patient (Spouse “Wife/Husband” = 1, Others = 0; Abdollahpour et al., 2012), how well caregivers get along with patients (Very Well = 1, Quite Well and Below = 0; Steadman et al., 2007), caregiving for others (Kim et al., 2019; Yes = 1, No = 0), and caregiving hours per week (Kim et al., 2012). We also controlled for differences in the education levels of the patient–caregiver dyad (Chiao et al., 2015): Reporting “junior college/polytechnic/diploma” or “university and above” was characterized as having high levels of education. All other responses were characterized as having low levels of education. The patient–caregiver dyad was defined as having different educational levels if responses differed based on these categories.

All analyses were conducted in STATA 15, and we used 90% confidence interval to evaluate statistical significance.

## Results

### Sample Size

This study included data from 311 patient–caregiver dyads recruited as part of the COMPASS study. However, 19 dyads did not answer questions regarding treatment preferences between pain management and life-extending treatment. An additional caregiver did not complete the caregiver reaction assessment, while six dyads provided incomplete information. A total of 26 dyads were therefore excluded, leaving the analytical sample with 285 patient–caregiver dyads.

### Patient and Caregiver Characteristics

Just over half of patients (52%) were female with a mean age of 61.70 ± 9.98 years. The majority of patients had lower levels of education (75%), were married (82%), were Chinese (76%), and had private insurance (65%). The average symptom burden reported among patients was 4.56 ± 4.60 (out of 36). Caregivers were younger (mean age = 49.08 ± 14.60), had higher levels of education (49%), and were predominantly female (65%). Most were also married (78%), Chinese (75%), and had at least adequate financial coverage for their monthly expenses (66%). Most had full-time or part-time jobs (60%), while half reported getting along very well with the patient (47%). About half (49%) were spouses and reported providing caregiving to someone else in addition to the patient. The average number of caregiving hours by the caregiver to the patient was 17.12 ± 19.97 h/week. About 42% of the patient–caregiver dyads differed in educational level with more caregivers having a higher level of education compared to patients (Table 1).

**Table 1.** Patient and Caregiver Characteristics (*N* = 285)

Variables	*N* (%)	Mean (*SD*)
*Patient characteristics*		
Female	149 (52)	
Age		61.70 (9.98)
High education: college/polytechnic/diploma/university and above (ref: low education—no formal education/primary/secondary/vocational/institute of technical education)	71 (25)	
Married (ref: separated/widowed/divorced/never married)	233 (82)	
Chinese (ref: Malay/Indian/others)	217 (76)	
Availability of private insurance (ref: no private insurance)	186 (65)	
Symptom burden (out of 36)		4.56 (4.60)
*Caregiver characteristics*		
Female	185 (65)	
Age		49.08 (14.60)
High education: college/polytechnic/diploma/university and above (ref: low education—no formal education/primary/secondary/vocational/institute of technical education)	140 (49)	
Married (ref: separated/widowed/divorced/never married)	222 (78)	
Chinese (ref: Malay/Indian/others)	215 (75)	
Adequate financial coverage (ref: not adequate)	187 (66)	
Working (full-time/part-time) (ref: not working)	172 (60)	
Got along very well with the patient (ref: quite well and below)	134 (47)	
Relationship to patient (spouse) (ref: others)	140 (49)	
Provided caregiving to someone else (ref: no)	139 (49)	
Number of caregiving hours		17.12 (19.97)
*Joint (patient and caregiver) characteristics*		
Different education levels (ref: same education levels)	121 (42)	

*Notes:* SD = standard deviation. Means presented are unadjusted means.

Overall, caregivers reported a mean of 2.97 ± 1.19 (out of 5) score for financial burden, 2.79 ± 0.81 (out of 5) for impact to schedule and health, and 2.35 ± 0.58 (out of 5) for lack of family support. Impact of finances resulting from caregiving was the greatest contributor to perceived caregiver burden. They also reported a mean score of 4.01 ± 0.57 (out of 5) for caregiving esteem (Table 2).

**Table 2.** Caregiver Reaction Assessment Scores (CRAS)

CRAS factor*a*	All dyads, *N* = 285	Concordant dyads, *n* = 115	Discordant dyads, *n* = 170	*p*
Impact on finances (out of 5)	2.97 (1.19)	2.84 (1.18)	3.05 (1.19)	.144
Lack of family support (out of 5)	2.35 (0.58)	2.25 (0.53)	2.42 (0.60)	.015
Caregiving esteem (out of 5)	4.01 (0.57)	4.10 (0.55)	3.95 (0.58)	.040
Impact on schedule and health (out of 5)	2.79 (0.81)	2.66 (0.77)	2.88 (0.83)	.025

*Notes:* Standard deviations in brackets; *p* value for *t*-test of differences between means.

^a^Higher scores on the Impact on Finances, Lack of Family Support, and Impact on Schedule and Health subscales indicate negative caregiving effects. A higher score on the Caregiving Esteem subscale indicates positive caregiving effects.

### Treatment Preferences and Patient–Caregiver Discordance

Overall, 39% of patients preferred focus of care to be life extension, while 38% preferred moderate life extension and symptom management; 24% preferred focus of care to be symptom management. Conversely, 23% of caregivers preferred focus of care to be life extension, 57% had moderate stance, while 20% preferred focus of care to be symptom management (Table 3).

**Table 3.** Treatment Preferences Among Patient–Caregiver Dyads

	Preferences for all dyads, *N* = 285		Preferences among discordant dyads, *n* = 170 (60%)		
Treatment preferences	Patient	Caregiver	Patient	Caregiver	Preferences among concordant dyads, *n* = 115 (40%)
Focus of care: symptom management	67 (24%)	57 (20%)	49 (29%)	39 (23%)	18 (16%)
Moderate life extension and symptom management	108 (38%)	162 (57%)	43 (25%)	97 (57%)	65 (57%)
Focus of care: life extension	110 (39%)	66 (23%)	78 (46%)	34 (20%)	32 (28%)

A significant number (60%) of the participating patient–caregiver dyads reported discordant treatment preferences (Table 3). Among discordant dyads, most caregivers reported preferring moderate life extension and symptom management (57%). Of caregivers, 23% preferred focus of care to be symptom management while 20% preferred the focus of care to be life extension. However, among discordant dyads, almost half of the patients preferred focusing on life-extending treatment (46%), while 29% preferred focusing on symptom management and 25% had a moderate stance.

Among concordant dyads, most preferred moderate life-extending treatment and symptom management (57%); 16% preferred focus of care to be symptom management while 28% preferred focus of care to be life extension.

Though the majority (85.26%) of patients reported having discussed treatment and care preferences with their family, only 10.88% reported having any Advance Care Plans. The proportions of patients discussing treatment preferences with family (49.82% vs. 35.44%) or having advance care planning (5.96% vs. 4.91%) were not significantly different among dyads having discordance compared to those having concordance.

### Treatment Preference Discordance and Perceived Caregiver Burden

Based on the *t*-test of means, discordant caregivers reported lower caregiving esteem compared to concordant caregivers (*p* = .04). Discordant caregivers also reported a higher perceived caregiver burden resulting from the impact on schedule and health (*p* = .03) and lack of family support (*p* = .02; Table 2).

In the multivariable analyses, patient–caregiver discordance in treatment preferences was associated with higher perceived caregiver burden relating to the impact on caregiver schedule and health (β = 0.16, *p* = .07) and lack of family support (β = 0.13, *p* = .04). Patient–caregiver discordance was also associated with higher perceived caregiver burden relating to “impact on finances” (β = 0.16, *p* = .21) and lower caregiving esteem (β = −0.09, *p* = .17), but these relationships were not statistically significant at the 10% level (Table 4).

**Table 4.** Ordinary Least Squares Regressions: Association Between Discordance and Caregiving Reaction Assessment Scale Domains

	Impact on schedule and health		Impact on finances		Lack of family support		CG self-esteem	
Variables	*Β* (*SE*)	*p*	*Β* (*SE*)	*p*	*Β* (*SE*)	*p*	*Β* (*SE*)	*p*
Discordance	0.16* (0.09)	.07	0.16 (0.13)	.21	0.13** (0.07)	.04	−0.09 (0.07)	.17
*Patient characteristics*								
Patient age	−0.01** (0.01)	.02	0.00 (0.01)	.80	0.00 (0.00)	.56	0.00 (0.00)	.22
Patient has insurance (ref: patient has no insurance)	0.03 (0.10)	.75	−0.24* (0.15)	.10	0.01 (0.07)	.92	−0.06 (0.08)	.46
Patient symptom burden	0.01 (0.01)	.59	−0.01 (0.02)	.58	−0.01 (0.01)	.32	0.00 (0.01)	.71
*Caregiver characteristics*								
Caregiver age	0.00 (0.01)	.75	−0.01 (0.01)	.19	0.00 (0.00)	.94	0.00 (0.00)	.41
Caregiver male (ref: female)	−0.01 (0.10)	.96	0.32** (0.14)	.02	0.00 (0.07)	.99	0.07 (0.07)	.33
Married (ref: separated/widowed/divorced/never married)	−0.19 (0.13)	.16	0.15 (0.18)	.39	−0.10 (0.09)	.27	0.11 (0.09)	.23
Financial adequacy: adequate and more than adequate (ref: do not know/usually inadequate/occasionally adequate)	−0.23** (0.10)	.02	−0.85*** (0.14)	.00	−0.23*** (0.07)	.00	0.09 (0.07)	.22
Employed full-time/part-time (ref: not working/retired/homemaker)	0.08 (0.10)	.41	0.22 (0.15)	.15	0.06 (0.08)	.47	−0.07 (0.07)	.31
Patient is the spouse (ref: other)	0.17 (0.15)	.26	0.25 (0.21)	.22	−0.01 (0.10)	.95	−0.04 (0.09)	.70
Caregiver gets along with patient very well (ref: not at all well/a little bit well/quite well)	−0.24*** (0.09)	.01	−0.15 (0.13)	.27	−0.22*** (0.07)	.00	0.39*** (0.07)	.00
Caregiving hours	0.01*** (0.00)	.00	0.00 (0.00)	.31	0.00 (0.00)	.87	0.00 (0.00)	.97
Caregiver cares for others	0.14 (0.09)	.14	0.05 (0.14)	.69	0.15** (0.07)	.03	0.03 (0.07)	.62
*Joint characteristics (patients and caregivers)*								
Different education levels (ref: same education levels)	−0.03 (0.11)	.78	0.05 (0.16)	.75	−0.11 (0.07)	.11	0.04 (0.07)	.59
Observations	285		285		285		285	

*Notes:* * indicates significance at 10% level, ** indicates significance at 5% level, and *** indicates significance at 1% level.

## Discussion and Implications

This study examined the associations between patient–caregiver dyad discordance regarding end-of-life treatment preferences, perceived caregiver burden, and caregiving esteem. Overall, results indicated that most dyads (60%) reported discordant treatment preferences. Discordance was also associated with higher perceived caregiver burden and lower caregiving esteem. However, these associations were significant for only two out of four domains of the CRAS (caregiver burden arising from the impact on caregiver schedule and health and lack of family support).

### Patient–Caregiver Treatment Preference Discordance

In line with past literature, we found that a significant number of patient–caregiver dyads reported discordant treatment preferences (60%). However, how discordance arose differed significantly. The caregivers in our study leaned toward a balance between life extension and symptom management (57%), while patients noted a preference toward life extension (46%). These findings contrast those reported in previous literature, where it was caregivers who preferred more life-extending treatments than chosen by the patients themselves (Ozdemir et al., 2019; Tang et al., 2005). These findings are unexpected, and we posit that they may stem from a lack of treatment care discussions among all the parties involved (i.e., the patient, their caregiver, and the physician). In support of this argument, we found that though 86% of patients reported discussing treatment care preferences with their family, only 11% reported having Advanced Care Plans, suggesting that important (and necessary) conversations regarding end-of-life care may still be lacking. If it is a lack of communication between the dyads that are driving discordance, it seems crucial that a greater focus be placed on facilitating important end-of-life discussions between patients and their caregivers. Efforts must also be made toward ensuring caregivers better understand where patient preferences lie and underlining the importance of preparing members of the dyad for any eventualities. This can likely be achieved through the development of interventions specifically targeted toward enhancing communication skills between patients and their caregivers and promoting end-of-life discussions. We expect that the results of our study can help with these developments.

### Associations Between Treatment Preference Discordance Among Patient–Caregiver Dyads and Perceived Caregiver Burden

As hypothesized, treatment preference discordance was significantly associated with increased impact on caregivers’ schedule and health, a dimension of caregiver burden. There may be several reasons for these correlations. First, when faced with disagreements regarding medical treatments, caregivers may begin to doubt their ability to advocate on the patient’s behalf, which may have an adverse impact on caregiver’s health. Second, there may also be increased perceived interference with their daily lives if they feel that those under their care are acting against their own best interests. These findings support an existing body of research indicating the importance of psychological therapy not only for patients, but also to address the psychological distress that many caregivers face due to the responsibilities of informal caregiving (Waldron et al., 2013). Results also underline the importance of considering the “unit of care” to be all those involved (i.e., the dyad) rather than just the patient.

We also noted significant associations between treatment preference discordance and perceived increased caregiver burden as a result of lack of family support. These results should also be understood within the context of Asian familial values. Namely, when a member of the family is ill, Asian families are expected to contribute to the decision-making and caregiving process (Ho et al., 2010). When there is a perceived lack of support from other family members, caregivers may begin to feel isolated and unprepared for the increased responsibility that comes as a result of being a surrogate decision maker and primary caregiver—feelings of distress that may have been exacerbated due to the added pressures of discordance. It is also important to note that a perceived lack of familial support may not necessarily indicate that other members of the family unit are not contributing to patient caregiving, simply that the caregiver does not feel that the support given is sufficient. This lends credence to our argument regarding the necessity of developing therapeutic interventions targeted at improving communication among patients and their caregivers.

Our results also indicated that two of the four CRAS dimensions (impact of finances and caregiving esteem) were not significantly correlated with treatment preference discordance. However, this is not to say that the perceived impact of the financial cost was not burdensome. In fact, for *all* dyads (both concordant and discordant), self-reported financial burden resulting from caregiving was observed to be the greatest contributor to perceived caregiver burden. While financial burden in this study was self-reported and may therefore not be reflective of actual burden, these results are important as they suggest that even with the subsidies offered by the Singapore government, out-of-pocket costs for chronic illnesses like cancer may still be associated with significant psychological distress for caregivers, pointing to a need for financial planning programs to be included in cancer treatment programs.

Similarly, while caregiver esteem was not significantly associated with discordance, caregivers reported high levels of perceived caregiving esteem. This may be because in Asian culture, caring for family members is seen as a duty (Ho et al., 2003). Under this context, it is possible that though caregiving may have a negative impact on caregiver health, lifestyle, and finances, caregivers might have perceived it as the right and dutiful course of action, thus increasing caregiving esteem.

### Limitations and Future Research

Despite the importance of our findings, they should be understood in the context of several limitations. First, we used cross-sectional data and thus cannot infer causality. Second, because we used a convenience sample of advanced cancer patients, results may not be generalizable to all advanced cancer patients in the country or patients with other terminal diseases. Third, as our sample consists primarily of Asians, findings also may not be generalizable to people from other cultural backgrounds where family values do not feature as prominently. For a better, more complete understanding of how treatment preference discordance is correlated with perceived caregiver burden, future studies should examine the noted associations cross-culturally. This study also only examined select dimensions of treatment preferences (e.g., life extension vs. symptom management) and how these may be correlated with patient–caregiver discordance. However, given that discordance is a complex factor that can be caused by numerous variables, other studies should consider the inclusion of more treatment preference dimensions.

### Implications

Theoretically, the findings of this study provide a better understanding toward how dyad discordance regarding treatment preferences may worsen the burdens felt by caregivers, and importantly, which aspects of their lives are most adversely affected. Practically, our findings aid, and support, the establishment of therapeutic interventions targeted toward improving communication skills and end-of-life discussions among patients and their caregivers. Our results also support the importance of treating the dyad as a unit of care, and the development of therapy programs that are specifically targeted toward resolving the burden felt by informal caregivers.

## Conclusions

By examining treatment preference discordance among patient–caregiver dyads and its associations with various perceived caregiver burden dimensions, this study highlighted several important findings. First, while there was significant treatment preference discordance among the dyads in our study, they resulted from caregivers’ preferences for moderate treatment options and patients’ preferences toward life-extending treatment. This may stem from insufficient communication and care discussions among patients, their caregivers, and their physicians, suggesting that a greater focus should be placed on properly communicating treatment options and facilitating discussions between those involved. Our results also underlined particularly vulnerable perceived caregiver burden dimensions (i.e., impact on caregiver schedule and health and lack of family support) that can be the focus of clinicians when discussing care pathways with patients and their caregivers. Therapy specifically targeted toward caregivers should also be developed to resolve the burden experienced by this population.
